# An Atypical Lipomatous Tumor of the Hypopharynx: Case Report

**DOI:** 10.7759/cureus.23348

**Published:** 2022-03-21

**Authors:** Mohammad Al-Kadi, Salman AlOtieschan, Mohammad Jihad Almahdi, Rafeef AlHajress

**Affiliations:** 1 Division of Otolaryngology - Head & Neck Surgery, Department of Surgery, King Abdulaziz Medical City, Riyadh, SAU; 2 Medicine, College of Medicine, King Saud Bin Abdulaziz University for Health Sciences, Riyadh, SAU; 3 Otolaryngology - Head & Neck Surgery, King Abdulaziz University Hospital, Riyadh, SAU

**Keywords:** hypopharyngeal tumors, piriform sinus, atypical spindle cell/pleomorphic lipomatous tumor, endoscopic approach, liposarcoma

## Abstract

Nearly 20% of all mesenchymal tumors are liposarcoma in origin, mostly occurring in extremities and trunk. However, few cases of liposarcoma in the hypopharynx have been documented. This atypical localization of liposarcoma warrants a great interest in reporting such a case. Here, we report an 81-year-old Saudi male who presented to our clinic complaining of progressive dysphagia and globus sensation for two months. On examination, using a flexible nasopharyngoscopy, a hypopharyngeal mass occupying the left piriform sinus originating from the mucosa of the posterior pharyngeal wall and anteriorly from the anterior and medial piriform sinus mucosa was observed. Contrasted head and neck CT-scan revealed a benign-looking well-defined left-sided submucosal cyst aligned along the left aryepiglottic fold encroaching and narrowing the laryngeal inlet with dimensions of 1.8×2.1×2.7 cm. The mass was resected successfully using a trans-oral approach. A histopathological review showed spindle stromal cells that reacted positively for CD34 (Qbend10) on immunohistochemical staining and positive result for MDM2 (12q15) Amp. The pathology result indicates an abnormal amplification of the MDM2 gene region. The patient was followed for almost two years without evidence of recurrence. In conclusion, atypical lipomatous tumors (ALTs) of the hypopharynx are rarely diagnosed, and the gold standard for diagnosis is biopsy. Transoral endoscopic approach has a better outcome than cervical approach. Follow-up of patients with ALT is crucial, due to the highly recurring nature of the disease. Here we present a rare case of ALT, the patient had complete remission without complication.

## Introduction

Liposarcoma is a malignant tumor of adipose tissue, most commonly seen in the extremities, trunk and retroperitoneum [1-2]. Rarely, liposarcomas are found in the head and neck, and when they do occur there, they account for 3-8% of cases [1,3]. Several classification systems have been adopted for liposarcoma, the most common classification system used is the World Health Organization (WHO) classification of liposarcoma [4-5]. WHO classified liposarcoma into four subtypes: Atypical lipomatous tumor (ALT)/well-differentiated liposarcoma, myxoid/round cell liposarcoma, dedifferentiated liposarcoma, and pleomorphic liposarcoma [4-5].

ALT is a well-differentiated type of liposarcoma that has a favorable outcome if treated surgically [3]. Liposarcoma of the hypopharynx is an unusual entity and very few cases have been reported in the literature. It is worth noting that ALT can present clinically as a Giant Fibrovascular Polyp (GFVP), which is a rare benign tumor that usually occurs in the esophagus and to a lesser extent in the hypopharynx [6].

ALT is differentiated from GFVP histologically, they present as variable size adipocytes, atypical multivacuolated lipoblast, and nuclear atypia in adipocytes and spindle cells [6]. The importance of differentiating between ALT and GFVP is due to the malignant nature of ALT, thus, ALT requires surgical intervention and must be closely followed up post-operatively as ALT has the tendency to recur [6]. Here we report the clinical and pathological findings of a rare case of atypical lipomatous tumor of the hypopharynx that was successfully treated surgically without evidence of recurrence.

## Case presentation

Here we present a case of an 81-year-old male, a former soldier at the Saudi National Guard, who presented to King Abdulaziz Medical City (KAMC) in Riyadh, Kingdom of Saudi Arabia. He was referred to our service complaining of difficulty swallowing solid food for the past two months, with a progressive deteriorating course. He chokes on food residue and complains of a bothersome globus sensation. The patient has a negative history of pneumonia, dysphonia, structural pharyngeal dysphagia, and B-symptoms (night sweats, fever, or weight loss). Past medical history was significant for hypothyroidism, hypertension, heart failure, anteroseptal MI, dyslipidemia, reflux esophagitis, gastric ulcer, right ear hearing impairment and chronic kidney disease.

**Figure 1:**
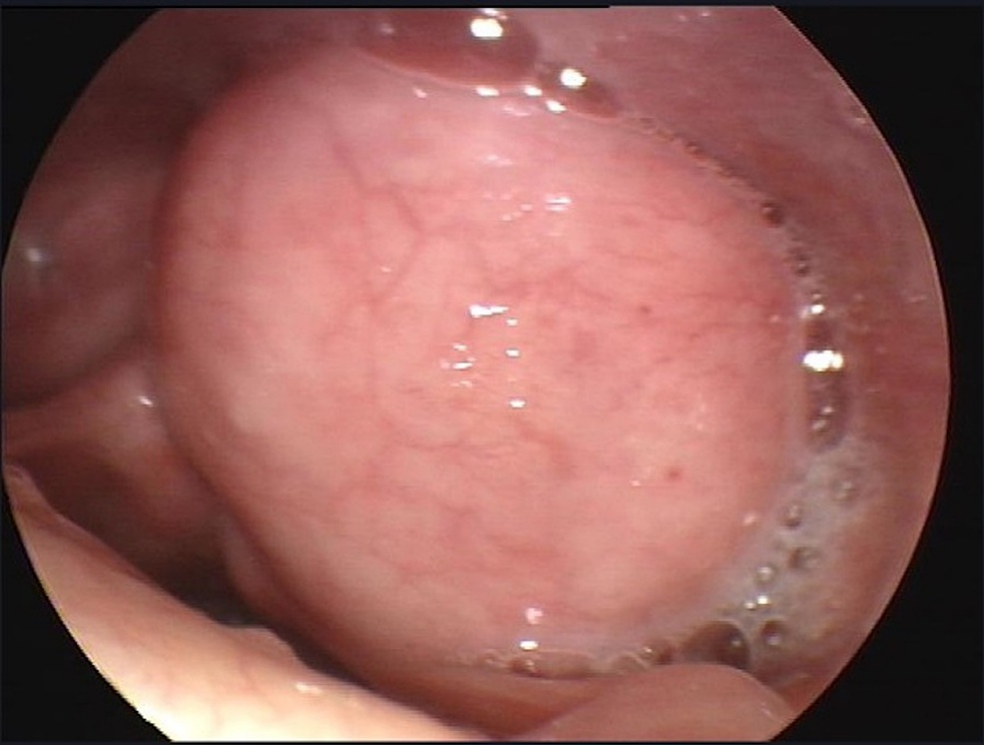
Pre-Op. Benign-looking well-defined left-sided submucosal hypopharyngeal cyst.

On physical exam using nasopharyngoscopy, a hypopharyngeal mass was seen occupying the left piriform sinus originating from the mucosa of the posterior pharyngeal wall and anteriorly from the anterior and medial piriform sinus mucosa (Figure 1). The mass was firm, almost 3 cm in diameter with no drainable material. The neck was soft with no evidence of cervical lymph node involvement. The trachea was patent and not shifted, and vocal cords were mobile bilaterally. No evident extension to any adjacent structure was appreciated at this stage.

**Figure 2:**
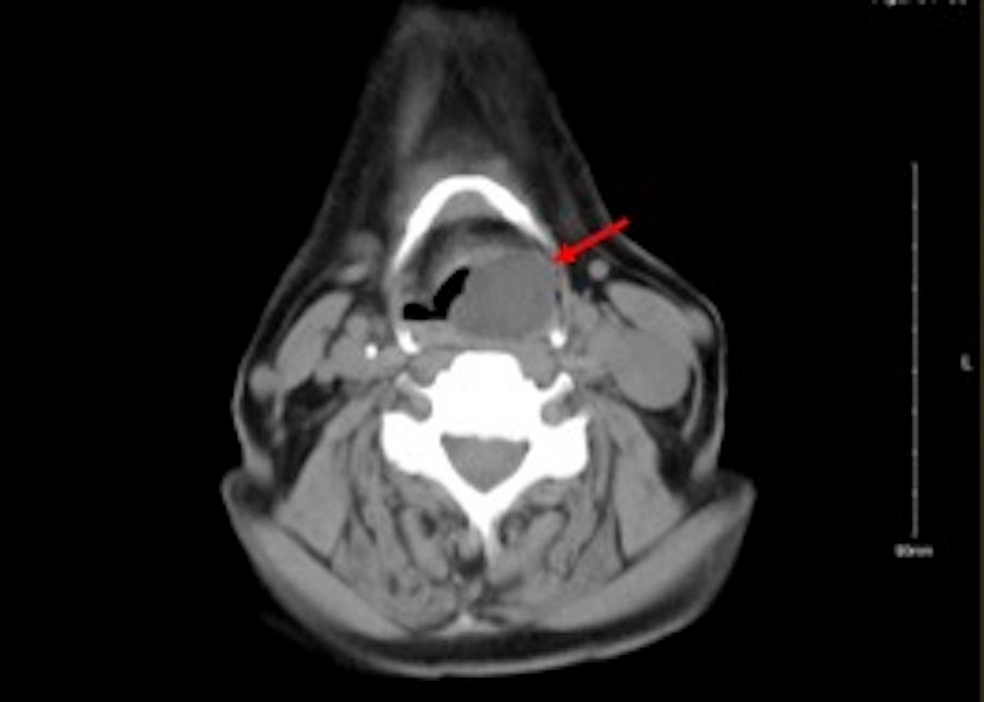
Pre-Op CT scan. Left-sided hypopharyngeal mass with a fatty core.

Further investigation with a contrasted head and neck CT-scan revealed a benign-looking well-defined left-sided submucosal cyst aligned along the left aryepiglottic fold encroaching and narrowing the laryngeal inlet with no extension beyond the pharyngeal wall. The mass’ appreciated dimensions were 1.8×2.1×2.7 cm (Figure 2).

There were no appreciable neck soft tissue masses or involvement of lymphoid tissues, neck vasculature was patent and there were no aggressive osseous lesions.

**Figure 3:**
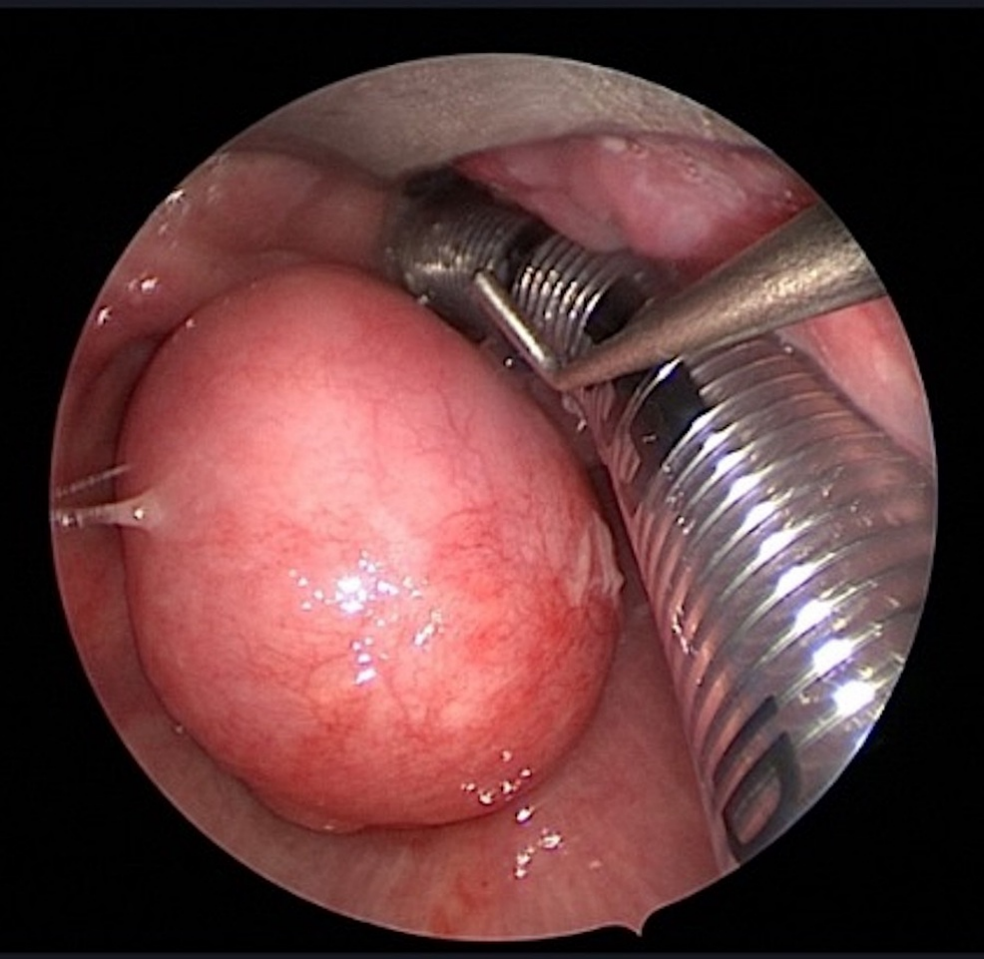
Intra-Op. Well-defined submucosal hypopharyngeal cyst.

Since the patient was symptomatic with a progressive complaint, we consented with him for primary surgical intervention of a complete transoral excision of the lesion. Intraoperatively and prior to the resection of the mass, examination showed findings similar to the previously mentioned (Figure 3).

**Figure 4:**
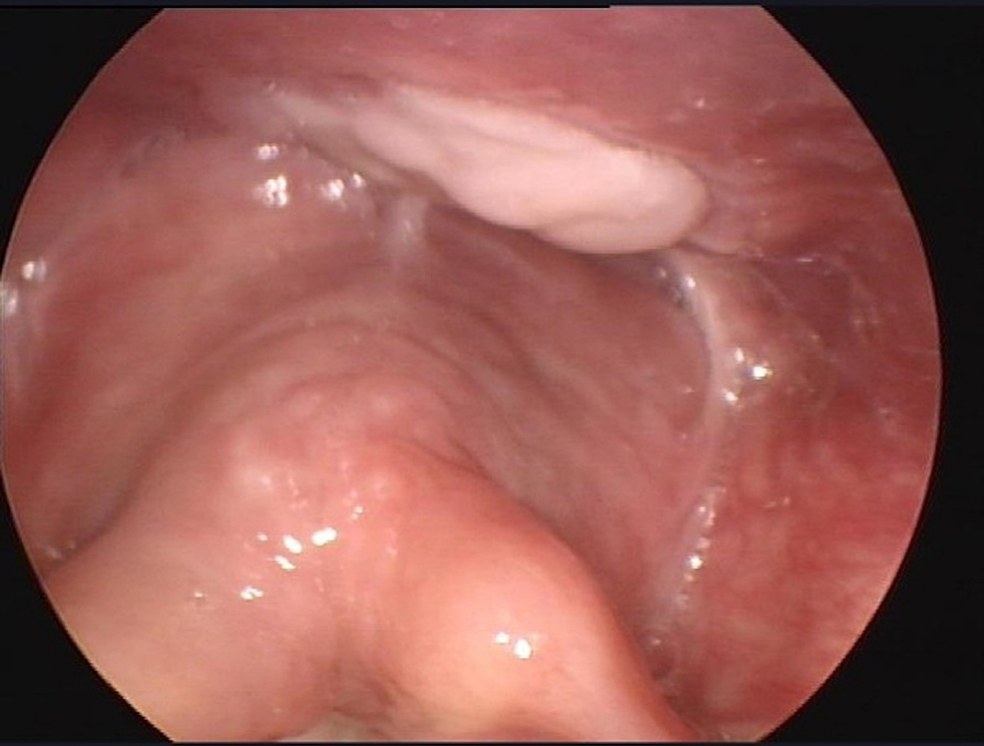
Post-Op. Healing with slough tissue appreciated at the site of the resected mass.

The mass was resected trans-oral with endoscopic assistance. Our approach was initially through an adult size Lindholm laryngoscope, however, we found a Boyle-Davis mouth gag could provide us a larger field and easies manipulation due to the limitation using the laryngoscope. The lesion was resected using laryngeal graspers and a monopolar to incise the mucosa and expose the mass and bipolar cautery to dissect it down to the stalk. On gross examination, the mass consisted of a tan-white, well-circumscribed soft tissue that measured 2.5×2.0×2.0 cm. Serial sectioning of the specimen revealed a glassy myxoid cut surface. No central necrosis or hemorrhage was observed. Histopathology report showed spindle stromal cells that react positively to CD34 (Qbend10) on immunohistochemical staining and positive result for MDM2 (12q15) Amp. This pathological result indicates an abnormal amplification of the MDM2 gene region. The patient was first followed up two weeks post-operatively, was recovering well and symptomatically improving, vocal folds’ mobility was not affected. There was no mass or lesion seen using a 70-degree rigid endoscope (Figure 4).

**Figure 5:**
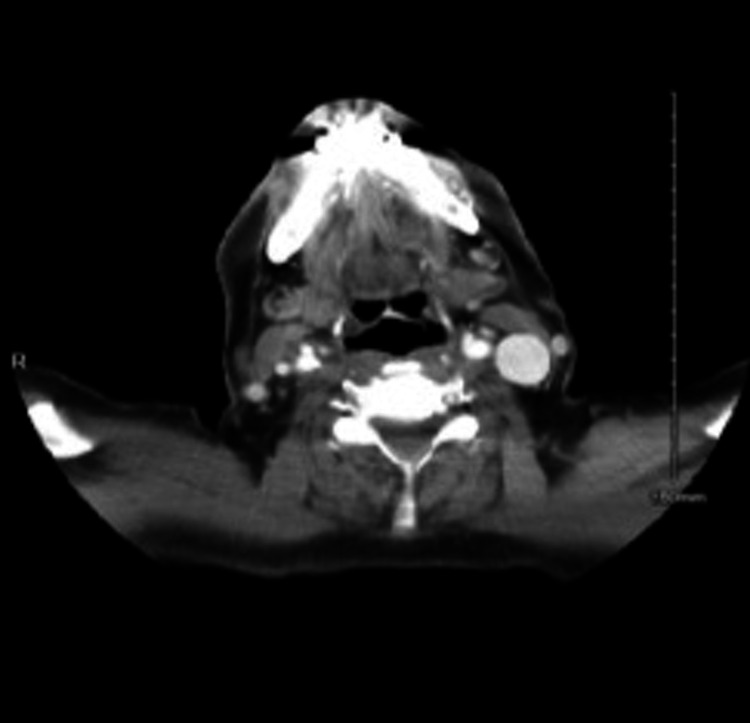
Post-Op CT scan. Clear hypopharynx with no sign of recurrence.

The patient was next seen four weeks later and was referred to radiation oncology who agreed on active surveillance due to the patient’s advanced age and comorbidities. Follow-up CT-scans of the head and neck (Figure 5), as well as the chest and abdomen, were all negative for metastasis and recurrence. The last clinical assessment of the patient was in April 2020. The patient was asymptomatic and no local recurrence was detected. The patient passed away in July 2020; not related to malignancy.

## Discussion

Liposarcomas are considered one of the most common soft tissues tumors in adults, with a reported incidence rate of up to 30% of all sarcomas [5,7-8]. Most cases of liposarcomas are found in the retroperitoneum, trunk and extremities [5,7-9]. In 1954, almost all liposarcoma cases reported were below the neck [10]. It was not until 1975 that the first case of laryngeal liposarcoma was reported by Miller et al. [5,7-9]. However, the localization of a liposarcoma in the hypopharynx is extremely rare [8]. Typically, patients with head and neck liposarcoma present in their 40-70s with a common manifestation of dysphagia [5,7-9]. Dysphagia usually begins with solid food and progresses to include liquids [8]. The progressive dysphagia observed in hypopharyngeal liposarcomas was similarly reported in patients with laryngeal liposarcomas, in addition to other similar clinical features [11]. A progressive disease course was observed in patients reporting weight loss and who appeared cachectic [8]. Furthermore, symptoms can manifest as airway obstruction, where large hypopharyngeal lipomatous mass can encroach the larynx as reported by Baj et al. [12].

Other clinical manifestations include, but are not limited to, regurgitation of a fleshy mass into the oropharynx or mouth, globus sensation, and odynophagia has also been reported [6]. The commonly used system to classify liposarcomas is the WHO classification system, which groups the tumor into four subtypes: Atypical lipomatous/well-differentiated, myxoid, pleomorphic and dedifferentiated liposarcomas [6,8]. Well-differentiated liposarcoma accounts for 30-40% of the cases and is the second most common subtype preceding dedifferentiated liposarcomas [6]. Diagnosis of liposarcoma can sometimes be challenging, due to clinical manifestation overlapping with other mass lesions such as lipoma, hamartoma, hemangioma, carcinoid tumor and specifically GFVP. Atypical liposarcomas can be distinguished from GFVP by histopathology [6].

In general, the diagnosis of hypopharynx liposarcoma involves clinical assessment using endoscopy, barium studies, CT-scan and histopathological analysis [6,8]. However, barium test is rarely used nowadays [6,8]. A CT scan is helpful in determining the size of the mass and the mass make-up, which shows a heterogenous mass with fat-like density [6,8]. The gold standard to diagnose liposarcomas of the hypopharynx is by biopsy. Macroscopically, the mass would present as soft, yellow, well-circumscribed and slowly growing mass covered by normal mucosa [6]. In addition, atypical lipomatous tumors have been associated with amplified MDM2 gene. The tumor size usually ranges from 3-15 cm [6].

The mainstay therapy of hypopharyngeal liposarcoma is surgical excision, followed by post-operative radiotherapy [6-12]. Excision is limited in the head and neck due to the proximity of neurovascular structures, and neck dissection is not indicated due to the low risk of cervical nodal metastasis [6]. The two surgical approaches used for hypopharyngeal liposarcoma are: cervical (lateral pharyngotomy) and endoscopic surgery [6,8]. The advantage of the transoral endoscopic approach over lateral pharyngotomy is the lower mortality rate, not requiring a tracheostomy, fast resumption of oral feeding 1-2 days post-operatively and shorter overall hospital stay [6]. In regards to the recurrence rate of atypical lipomatous tumors, Reed and Vick have reported an average of 69 months, thus, patients are indicated for active long-term follow-up and surveillance [13].

Here we represent a rare case of atypical lipomatous tumor of the hypopharynx that was treated surgically using a transoral endoscopic approach followed by active surveillance. The tumor was successfully removed with negative margins. The pathology report showed an amplification of the MDM2 gene, which is seen in atypical lipomatous tumor [6-8]. The patient recovered well as shown in the post-op endoscopy (Figure 4) and follow-up CT scan (Figure 5) - both showed complete remission and no sign of recurrence. During the follow-up period that stretched for two years, no recurrence was detected. The patient passed away in mid-2020, unrelated to hypopharyngeal liposarcoma or any of its complications.

## Conclusions

In conclusion, liposarcoma of the hypopharynx is a rare finding of liposarcomas in general. It has a less aggressive course and can easily be mistaken for other hypopharynx mass due to its rarity and similar appearance to other differentials. Definite diagnosis of an atypical lipomatous tumor is by biopsy followed by genetic testing for MDM2 and CD34. Transoral endoscopic approach is favored over lateral pharyngotomy (cervical approach) because it is associated with less surgical morbidities and complications (e.g., injury of spinal accessory nerve), decreased hospital stay and avoidance of tracheostomy. Though surgical complication can occur with transoral endoscopic approach, it occurs in lower frequency than in the trans-cervical approach. Follow-up of patients with ALT is crucial, due to the high recurrence nature of the disease. Here we present a rare case of ALT, in which the patient had complete remission without complication.
